# A new *Paraleius* species (Acari, Oribatida, Scheloribatidae) associated with bark beetles (Curculionidae, Scolytinae) in Canada

**DOI:** 10.3897/zookeys.667.12104

**Published:** 2017-04-10

**Authors:** Wayne Knee

**Affiliations:** 1 Canadian National Collection of Insects, Arachnids, and Nematodes, Agriculture and Agri-Food Canada, 960 Carling Avenue, K.W. Neatby Building, Ottawa, Ontario, K1A 0C6, Canada

**Keywords:** Mite, Acari, phoresy, COI, forest entomology, ecology

## Abstract

Bark beetles (Scolytinae) are hosts to a broad diversity of mites (Acari), including several genera of Oribatida (Sarcoptiformes). Of these, *Paraleius* (Scheloribatidae) species are the most frequently collected oribatid mites associated with bark beetles. A new species was discovered while surveying the acarofauna of bark beetles in Eastern Canada and is described as *Paraleius leahae* **sp. n.** (Oribatida, Scheloribatidae). This species was collected from two host beetle species, *Hylastes porculus* Erickson and *Dendroctonus valens* LeConte, in Ontario, New Brunswick and Nova Scotia. The genus *Paraleius* is rediagnosed, *Metaleius* is considered a synonym of *Paraleius*, and the proposed synonymy of *Paraleius* with *Siculobata* is rejected. The three known species are *Paraleius leontonycha* (Berlese), *P. leahae* **sp. n.**, and *P. strenzkei* (Travé), **comb. n.** The barcode region of cytochrome oxidase subunit I (COI) was amplified from *P. leahae* **sp. n.**

## Introduction

A broad assemblage of wood-burrowing beetles (Cerambycidae, Buprestidae, Scolytinae), and associated mites, nematodes, and fungi reside under the bark of dead, dying or living trees. Bark beetles (Curculionidae, Scolytinae) are a diverse group of wood-borers that feed and mate in the cambium or xylem of numerous tree species worldwide (Wood 1982). Mites are among one of the most diverse and common associates of scolytines, and in temperate forests some bark beetle species are associated with 15-20 mite species (Lindquist 1969).

Oribatid mites (Acari, Oribatida) dwell primarily in soil or forest litter, though many are found in arboreal habitats and a few occur in aquatic systems (Norton and Behan-Pelletier 2009). Several genera of oribatid mites are also found under tree bark in scolytine galleries, or dispersing phoretically on bark beetles (Moser and Roton 1971). Phoresy is relatively uncommon in oribatid mites, and typically phoretic species are not host specific and the association with their host appears to be passive (Norton 1980). *Paraleius leontonycha* (Berlese, 1910) (Scheloribatidae) is the most frequently encountered oribatid species on bark beetles, although it does not occur in high abundance or prevalence (Knee et al. 2013). *Paraleius leontonycha* is a broad host generalist with a Holarctic distribution, collected from 17 scolytine species and found in the galleries of 10 other bark beetle species (Knee et al. 2013, Ahadiyat and Akrami 2015). A recent survey of the mesostigmatic and oribatid mite fauna of bark beetles in eastern Ontario (Knee et al. 2013) uncovered a new species of the monotypic genus *Paraleius* Travé, 1960 associated with two scolytine species, *Hylastes porculus* and *Dendroctonus valens*. Herein, I propose and describe *Paraleius leahae* sp. n., including the barcode region of COI. I also provide a revised generic diagnosis for *Paraleius*, and resolve some taxonomic issues surrounding *Paraleius* and closely related genera.

## Methods

### Sampling and identifications

Bark beetle specimens collected with Lindgren funnel traps in eastern Ontario by Knee et al. (2013), and Eastern Canada by the Canadian Food Inspection Agency (CFIA) staff as part of the Invasive Alien Species Monitoring program, were examined for associated mites. Scolytines were identified to species using a dissecting microscope and keys from Bright (1976). The presence, abundance, and attachment location of oribatid mites was recorded. All mites were collected and preserved in 95% ethanol for later identification and/or molecular analysis. Specimens used for illustrations were mounted in Hoyer’s medium on temporary cavity slides. Permanent slide-mounted mites were cleared in 85% lactic acid, mounted in polyvinyl alcohol medium (6371A, BioQuip Products, Rancho Dominguez, California, United States of America), and cured on a slide warmer at 40ºC for 3–4 days.

Oribatid collections at the Canadian National Collection of Insects, Arachnids, and Nematodes (CNC), State University of New York College of Environmental Science and Forestry (SUNY-ESF), and John Moser’s collection at the United States Department of Agriculture (USDA) in Pineville, Louisiana were examined for *Paraleius* specimens.

Slide-mounted specimens were examined using a Leica DM2500 compound microscope and Leica ICC550 HD camera, with differential interference contrast illumination (DIC). Initial drawings of mites were made with pencil on paper using a camera lucida. These were later merged in Adobe Photoshop CS5 and redrawn in Adobe Illustrator CS5 using an Intuos 3 Graphics Tablet from WACOM Co., Ltd. (Saitama, Japan).

Morphological terminology used in this study follows that developed by F. Grandjean (see Travé and Vachon 1975 for references and Norton and Behan-Pelletier 2009 for overview). Notogastral setation follows the unideficient nomenclature detailed by R.A. Norton in Balogh and Balogh (1988). The following conventions for measurements are used: *prodorsal setae*, measured on permanent slide-mounted specimens (*ro*, rostral seta; *le*, lamellar seta; *in*, interlamellar seta; *ex*, exobothridial seta; *bo*, bothridial seta); *total length*, measured dorsally from tip of rostrum to posterior margin of the notogaster on specimens in cavity slides; *total width*, measured at widest part of the notogaster on specimens in cavity slides. Total length and width were measured only for the few mites that were stored in ethanol and not mounted on permanent slides, all other measurements were performed on five to seven slide mounted mites. Leg setation is presented as the number of setae per segment (including the famulus on tarsus I), with solenidial counts in parentheses, in the following order: trochanter–femur–genu–tibia–tarsus. All measurements are in micrometres (µm); lengths presented with mean followed by the range in parenthesis. Type specimens are deposited in the Canadian National Collection of Insects, Arachnids, and Nematodes, at Agriculture and Agri-Food Canada, Ottawa, Ontario, Canada.

### Molecular methods

Genomic DNA was extracted from whole specimens for 24 hours using a DNeasy Tissue kit (Qiagen, Inc., Santa Clara, California, United States of America). Following extraction, mites were removed from the extraction buffer, vouchers were-slide mounted, and genomic DNA was purified following the DNeasy Tissue kit protocol. PCR amplifications were performed in a total volume of 25 µl, with 14.7 µl ddH2O, 2.5 µl 10× ExTaq buffer, 0.65 µl 25 mM MgCl2, 1.0 µl of each 10 µM primer, 2.0 µl 10 mM dNTPs, 0.15 µl ExTaq DNA polymerase, and 3 µl genomic DNA template. Primer pairs PHF1 (5’–CWACAAAYCAYAAAGATATTGG–3’) and PHR1 (5’–TAHACYTCHGGRTGVCCRAAAAAYCA–3’) were used to amplify a 641 bp fragment of the 5’–end of COI. PCR amplification was performed on an Eppendorf ep Gradient S Mastercycler (Eppendorf AG, Hamburg, Germany), using the following protocol: initial denaturation cycle at 94 °C for 3 min, followed by 45 cycles of 94 °C for 45 s, primer annealing at 40 °C for 45 s, 72 °C for 1 min, and a final extension at 72 °C for 5 min. Amplified products and negative controls were visualized on 1% agarose electrophoresis gels, and purified using pre-cast E-Gel CloneWell 0.8% SYBR Safe agarose gels (Invitrogen, Carlsbad, California, United States of America). Sequencing reactions followed the protocol of Knee et al. (2012), and sequencing was performed at the Agriculture and Agri-Food Canada, Ottawa Research and Development Centre, Core Sequencing Facility (Ottawa, Ontario, Canada). Sequence chromatograms were edited and contiguous sequences were assembled using Sequencher v5.3 (Gene Codes Corp., Ann Arbor, Michigan, United States of America). Sequence for *Paraleius leahae* sp. n. has been submitted to GenBank (KY402259).

## Results and discussion

### Family Scheloribatidae Grandjean, 1933

#### Genus Paraleius

Taxon classificationAnimaliaSarcoptiformesScheloribatidae

Travé, 1960

##### Type species.

*Paraleius* (=*Oribella*) *leontonycha* (Berlese, 1910)

##### Revised diagnosis.

Rostrum extended medially, forming narrow point; anterior border of notogaster convex; prodorsal setae long, thickened, attenuate, barbed; bothridium inserted dorsolaterally, close to lamella; bothridial seta capitate or fusiform; bothridium covered with numerous spicules; prolamella present; sublamella and translamella absent; pteromorphs absent; exobothridial seta (*ex*) medium sized and barbed; humeral porose organ (*Ah*) expressed as saccule; four pairs of saccules on notogaster; Ten pairs of medium sized notogastral setae; shallow sternal groove on ventral surface; solenidia of tibiae III and IV microcephalic (rounded vesicle) or not; eupathidia *p* of tarsus I smooth, seta *p* of tarsus II–IV with small bristles along one side; seta *s* of tarsus I with large barbs along ventral side, not eupathidial; leg pretarsi monodactylous or hetero-tridactylous with large curved median claw, lateral claws (if present) long and thin, resembling setae.

##### Remarks.

Travé (1960) described *Paraleius* as closely resembling *Hemileius* Berlese, 1916 with the distinction of the following characters: rostrum extended medially, forming narrow point; bothridial seta capitate; sublamella absent; seta *ex* medium sized and barbed; *Ah* expressed as saccule; heterodactyl claws with pronounced central claw; solenidia of tibiae III and IV microcephalic. Travé’s diagnosis lacked a few additional characters which have been included in the revised diagnosis above: notogaster anterior margin convex, bothridium inserted close to the lamella, numerous spicules on bothridium. To accommodate the new species herein described the description for three character states from Travé’s original diagnosis were modified: bothridial seta shape, pretarsal dactyly, and solenidia of tibiae III and IV microcephalic or not.

While Weigmann (1969) treated *Paraleius, Metaleius* and *Siculobata* as distinct genera, he later (2006) considered *Paraleius* and *Metaleius* to be junior synonyms of *Siculobata* based on a shared lamellar complex. However, this complex is not identical: *Siculobata* has a rudimentary sublamella, while *Paraleius* and *Metaleius* lack a sublamella. The synonymization of these genera also overlooks several other distinct character states shared by *Paraleius* and *Metaleius* that *Siculobata* does not possess. These include: rostrum with narrow medial point, anterior margin of notogaster convex, seta *ex* medium sized and barbed, *Ah* expressed as saccule, and bothridial seta inserted dorsolaterally close to lamella. Fredes and Martinez (2013) did not follow Weigmann’s (2006) proposed synonymy and provided a diagnosis for *Siculobata* *sensu stricto* that excludes *Paraleius* and *Metaleius*. Based on their concepts and on the aforementioned shared character states, I also reject the synonymization of *Paraleius* with *Siculobata*, but synonymize *Metaleius* and *Paraleius*. Each of the latter genera has been monotypic to this point, so the revised diagnosis for *Paraleius* is based on *Paraleius leontonycha*, *Paraleius leahae* sp. n., and *Paraleius strenzkei* (Travé, 1960), comb. n.

In his checklist of the world oribatid mite fauna, Subías (2004) placed Wallworkiella Hammer, 1979 as a subgenus of Paraleius, with the single species Paraleius (Wallworkiella) nasalis (Hammer, 1979). No explanation or justification was provided by Subías. In an unpublished online update (Subías 2016), possibly following Weigmann’s classification, he instead placed *Wallworkiella* as a subgenus of *Siculobata*. However, *Wallworkiella* differs from *Paraleius* by having five pairs of notogastral sacculi, homo-tridactylous tarsi, and inflated tarsal pulvilli. Additionally, *Wallworkiella* does not belong to *Siculobata* based upon the concept of Fredes and Martinez (2013). Clearly, the generic and species level relationships of Scheloribatidae require further research and revisions, but the demotion of *Wallworkiella* to subgeneric rank under either *Paraleius* or *Siculobata* is unsupported.

#### Paraleius leahae sp. n.

Taxon classificationAnimaliaSarcoptiformesScheloribatidae

http://zoobank.org/1B2E5D72-E272-4867-BE09-58C7EB727B46

**Figure 1.**
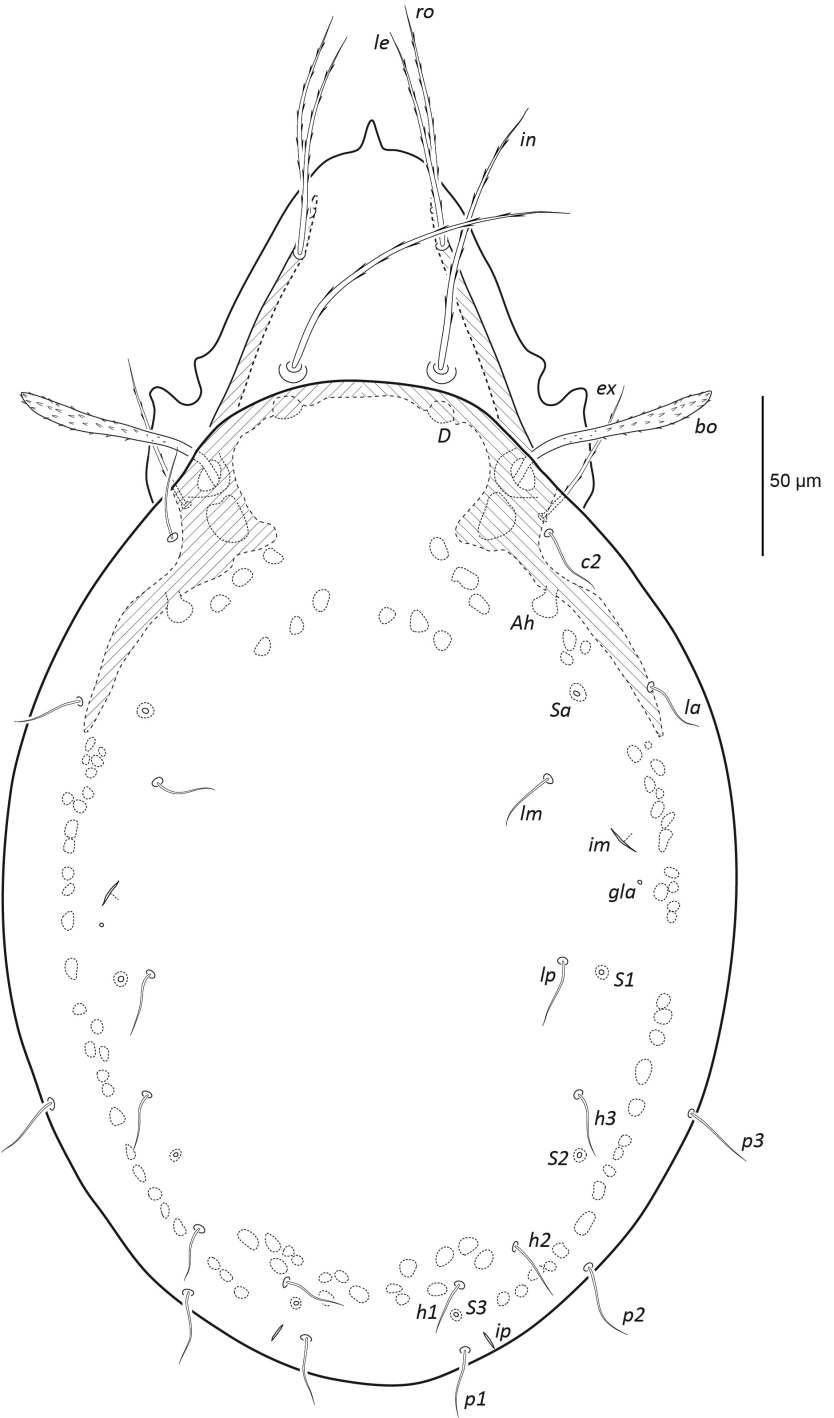
Female *Paraleius leahae* sp. n. dorsal view, legs omitted.

**Figure 2.**
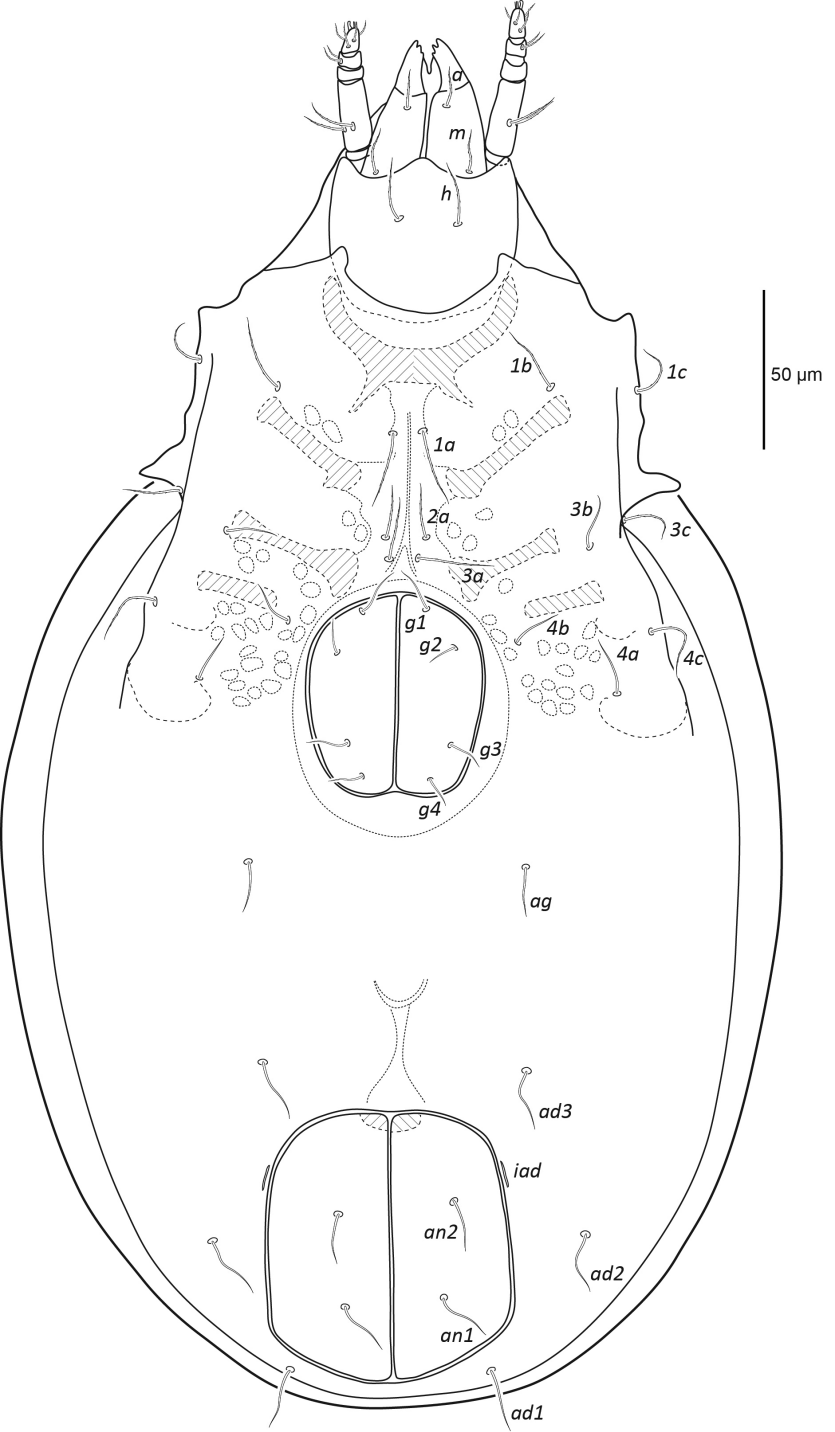
Female *Paraleius leahae* sp. n. ventral view, legs omitted.

**Figure 3.**
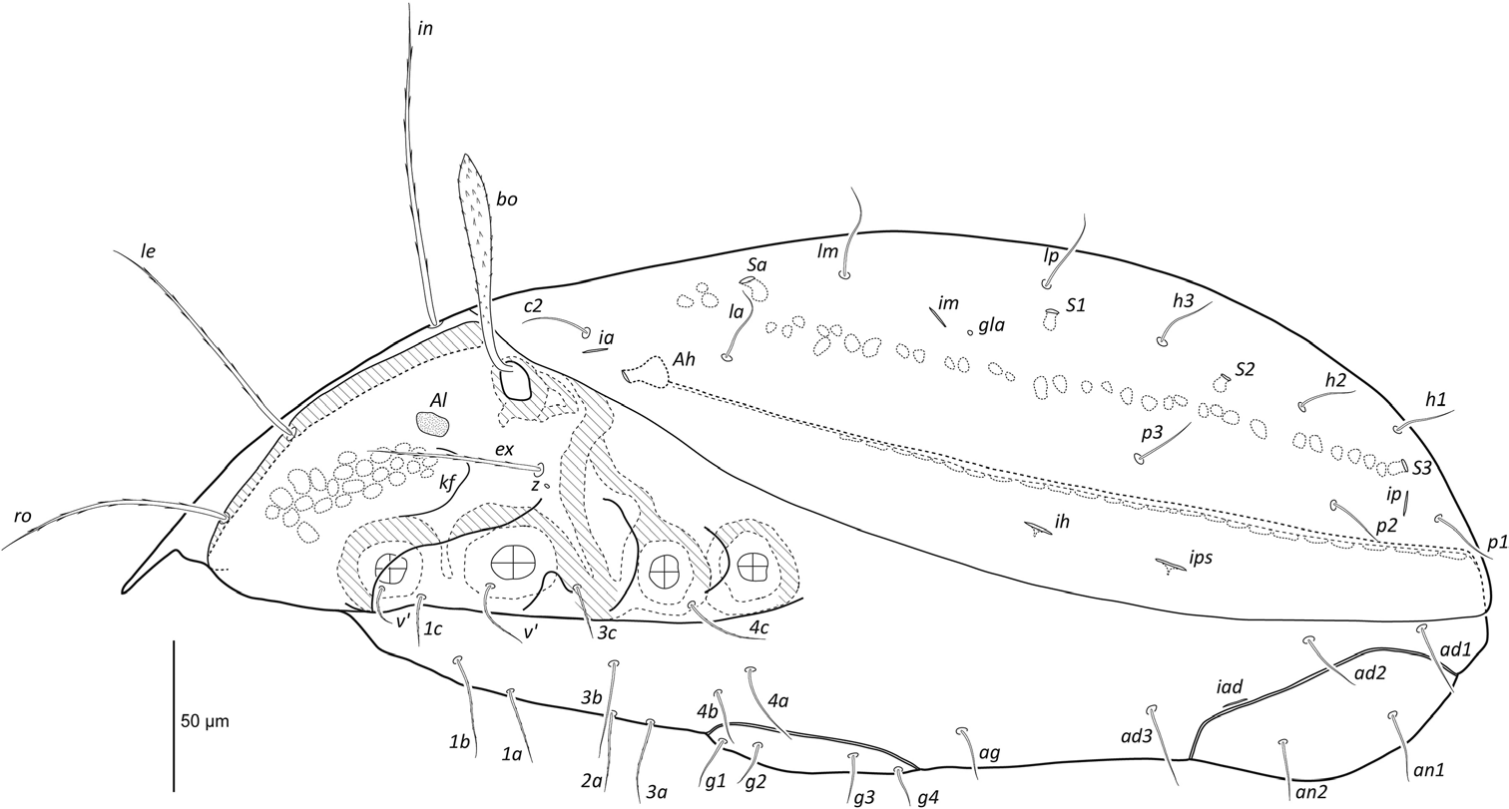
Female *Paraleius leahae* sp. n. lateral view, legs and gnathosoma omitted.

**Figure 4.**
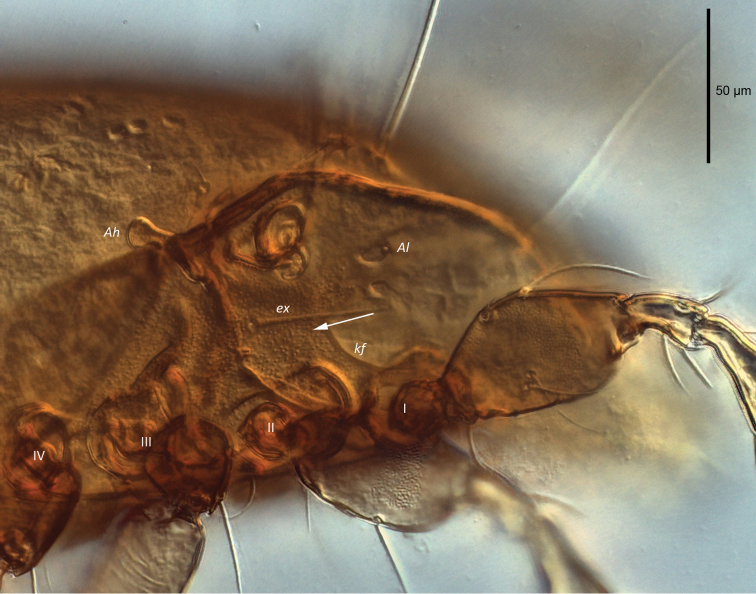
Female *Paraleius leahae* sp. n. photomicrograph of lateral view (DIC illumination), arrow indicating cuticular microtubercles.

**Figure 5.**
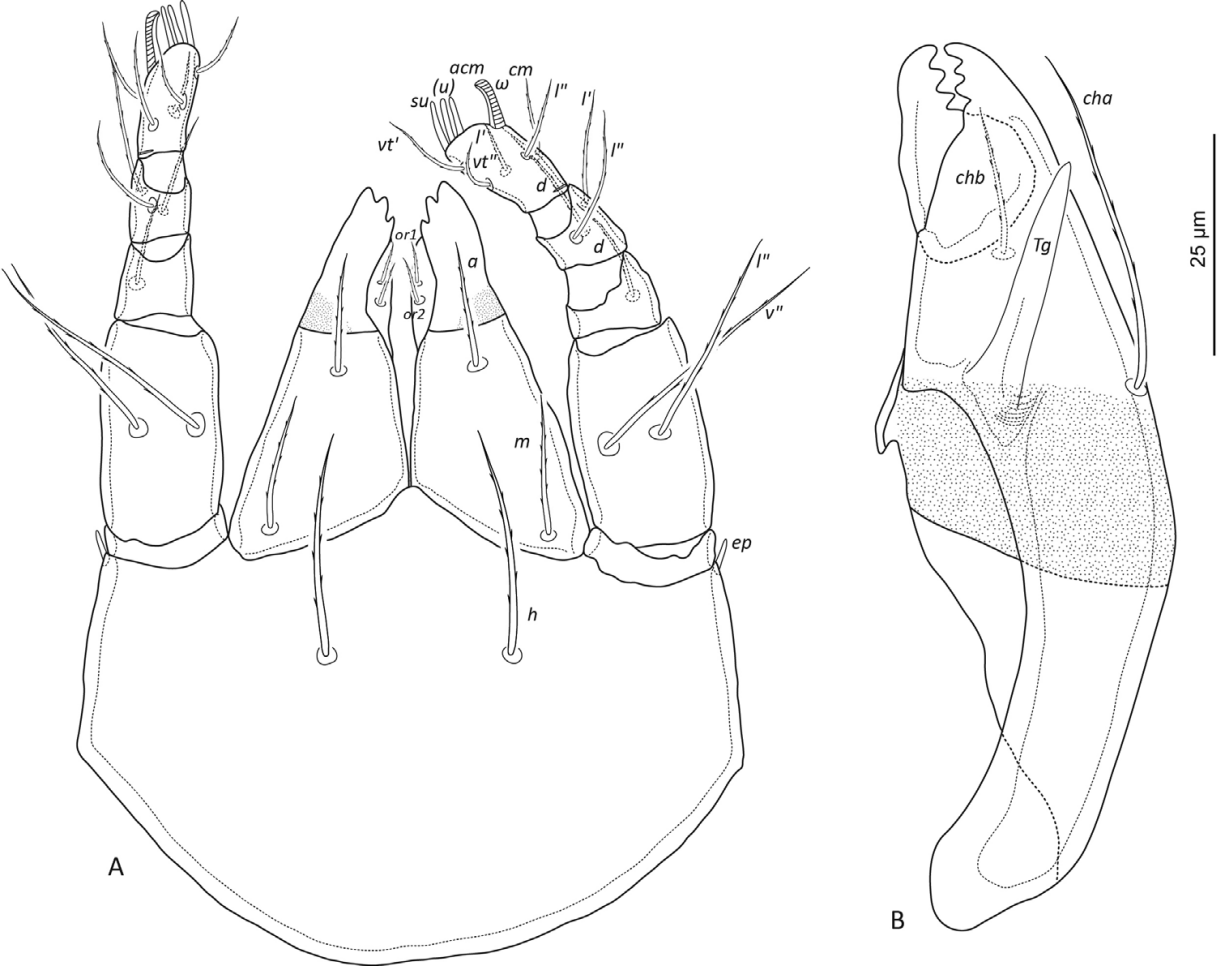
Female *Paraleius leahae* sp. n. **A** ventral view of subcapitulum **B** chelicerae, paraxial view.

**Figure 6.**
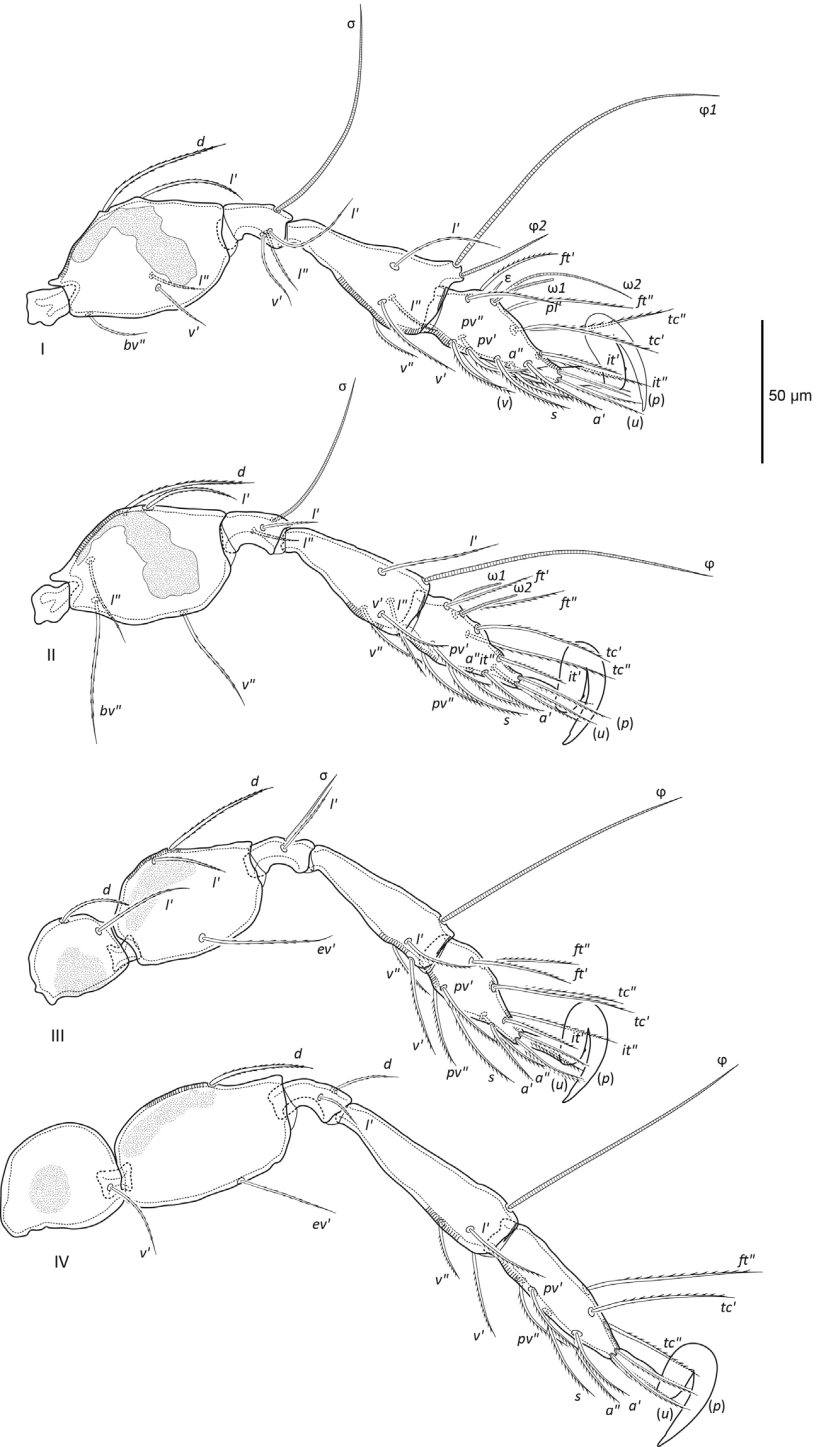
Female *Paraleius leahae* sp. n. legs; legs I, II paraxial view, legs III, IV antiaxial view.

Figs 1 , 2 , 3 , 4 , 5 , 6

##### Material examined.

***Type material*.** Holotype: adult female (vial CNC649357) on *Hylastes porculus* (female) collected in Westfield, Nova Scotia, Canada (44.40316, -64.97473), 28.v.2009, coll: W. Knee.

Paratypes (20): one female (vial CNC649359) with the same collection information as the holotype; female (vial CNC649361) on *H. porculus* (male), St. Stephen, Highway 1, New Brunswick (45.22321, -67.15371), 15.vi.2009, coll: W. Knee; female (vial CNC649362) on *H. porculus* (male), Bayside, Route 127, New Brunswick (45.20539, -67.14034), 15.vi.2009, coll: W. Knee; male (vial CNC649363) on *H. porculus* (female), Turner and Turner Mill, West Northfield, Nova Scotia, 1.vi.2009, coll: W. Knee; two females and two males (slides CNC649365–649368) on *H. porculus*, Algonquin Provincial Park (PP), Ontario (45.902, -77.605), 17.vi.2008, coll: W. Knee; one female and three males (slides CNC649371–649374) on *H. porculus*, Algonquin PP, Ontario (45.902, -77.605), 3.vi.2008, coll: W. Knee; two females (slides CNC649375, CNC649376) on *Dendroctonus valens*, Algonquin PP, Ontario (45.895, -78.071), 3.vi.2008, coll: W. Knee; three females and three males (slides CNC649378–649383) on *D. valens*, Algonquin PP, Ontario (45.895, -78.071), 28.v.2008, coll: W. Knee.

##### Other material.

67 slide mounted specimens from *D. valens*, and 22 from *H. porculus* collected in Algonquin PP, Ontario (45.895, -78.071), 2008–2009, coll: W. Knee; one slide mounted specimen from *D. valens*, and 70 from *H. porculus* collected in Algonquin PP, Ontario (45.902, -77.605), 2008–2009, coll: W. Knee.

##### Diagnosis adult.

As for *Paraleius* (see above). Bothridial seta long and fusiform, covered with numerous spicules; carina *kf* present; tarsi monodactylous with prominent sickle shaped strongly hooked claw; solenidia of tibiae III and IV not microcephalic. Immatures unknown.

##### Description.

***Measurements*.** Total length female (*n* = 4) 453 (432–464), male (*n* = 7) 430 (423–440). Total width female (*n* = 4) 277 (255–296), male (*n* = 7) 274 (258–295).

***Integument.*** Body cuticle red-brown. Notogastral surface and venter appear smooth, but with fine granulate structure at higher magnification (100x). Small microtubercles on epimeral surface (Fig. 4). Small microtubercles medially on subcapitulum between *h* setae.

***Prodorsum*** (Figs 1, 3). Lamella narrow, about 63 long. Prolamella narrow, extending from base of seta *le* to slightly anterior to *ro*, about 47 long. All prodorsal setae long, thickened, attenuate, barbed, reaching beyond rostrum; *ro* 78 (63–85) and *le* 95 (91–102) directed anteriorly, *in* 127 (121–137) directed anterodorsally. Mutual distance of setal pairs *ro*, *le*, and *in* ~53, 54, and 56 respectively. Bothridial seta long 85 (79–89), fusiform, directed anterolaterally, spicules conspicuous on head and minute along stalk. Seta *ex* medium sized 55 (52–59) thick, attenuate and barbed.

***Lateral aspect of podosoma*** (Figs 3, 4). Carina *kf* present. As for other scheloribatids pedotectum I large, visible from dorsal aspect. Pedotectum II smaller and less visible than pedotectum I. Circumpedal carina weakly curved, extending slightly posterior of acetabulum IV. Sublamellar porose area *Al* present. Humeral porose organ *Ah* (~14 length, 11 width) expressed as saccule. Gland opening *z* ventral to *ex*.

***Notogaster*** (Figs 1, 3). Longer than wide, ratio approximately 1.3:1. Dorsophragma (*D*) small, oval, approximately 8 wide. Ten pairs of medium sized notogastral setae 29 (19–38), setiform, smooth. Four pairs of saccules present: *Sa* largest (~9 diameter of saccule), located lateral to seta *la*; *S*_1_ (~7) lateral to *lp*; *S*_2_ (~6) posterolateral to *h*_3_; *S*_3_ (~6) posterior to *h*_1_. Lyrifissure *ia* posterolateral of seta *c*_2_; *im* posterolateral of *lm*; *ih* anterolateral to *p*_3_; *ips* posterolateral to *p*_3_; *ip* lateral to *p*_1_. Opisthonotal gland opening (*gla*) posterior of lyrifissure *im*.

***Venter*** (Figs 2, 3). Epimeral setal formula 3-1-3-3. All epimeral setae setiform and slightly barbed except for smooth setae *2b*, *4a*, and *4b*. Setal lengths as follows: *1a*, *1b*, *1c* ~26, 30, 25, respectively; *2a*, *3a*, *3b*, *3c* ~ 29, 28, 26, 26; and *4a*, *4b*, *4c* ~ 18, 22, 24. Shallow sternal groove present, approximately 69 long. Genital plates nearly as wide as long, genital plates of female slightly larger than those of male; length to width in females 61x58 and in males 55x51. Four pairs of simple setiform genital setae 14–21 long. Single pair of simple setiform aggenital setae (19), three pairs of simple setiform adanal setae *ad*_1_, *ad*_2_, *ad*_3_ ~24, 26, 27, and two pairs of simple setiform anal setae (23). Lyrifissure *iad* very close to anal plates, about midway between levels of setae *ad*_2_ and *ad*_3_.

***Gnathosoma*** (Fig. 5). Subcapitulum wider than long; porose region on rutelli. Subcapitular setae setiform, barbed, *h* (32), *m* (18), *a* (18). Adoral setae (*or*_1_, *or*_2_) thin and barbed. Palp with setation 0-2-1-3-9(1), palpal solenidion ω and seta *acm* fused (~9), slightly curved near tip. Postpalpal setae (*ep*) simple, smooth and rounded. Chelicera 108 long, setae attenuate barbed; *cha* (44), *chb* (18), Trägårdh’s organ (*Tg*) elongate triangular, rounded distally.

***Legs*** (Fig. 6; Table 1). All tarsi monodactylous with prominent sickle shaped strongly hooked claw, claw surface smooth except for small bump along inner margin. Large porose areas present on femora I–IV, and on trochanters III and IV. Ventral porose region present distally on tibiae I–IV and proximally on tarsi I–IV; dorsal porose area present distally on tarsi I–IV. Setal formula same as *P. leontonycha*. Leg setation (solenidia) of leg I: 1–5–3(1)–4(2)–19(2); II: 1–5–2(1)–4(1)–15(2); III: 2–3–1(1)–3(1)–15; IV: 1–2–2–3(1)–12 (Table 1). All setae on trochanters and genua I–IV barbed. Seta *l*’ on tibiae I, II barbed, all other setae on tibiae and tarsi I–IV with large barbs on one side, ventral setae with noticeably longer barbs than dorsal setae. Eupathidia *p* of tarsus I (~27), setae *p* of tarsi II–IV and *u* of tarsi I–IV with slight barbs unilaterally on ventral side. Famulus (8) short and blunt distally. Solenidia ω_1_ on tarsus I baculiform, ω_1_ and ω_2_ on tarsus II ceratiform, all other solenidia piliform. Solenidia of tibiae III and IV not microcephalic. Bases of solenidia ω_1_ and ω_2_ on tarsus I positioned very close together.

**Table 1.** Leg setation and solenidia of adult *Paraleius leahae* sp. n., single prime (’) indicates setae on anterior and double prime (”) setae on posterior, seta in parenthesis indicates the presence of both setae.

Leg	Trochanter	Femur	Genu	Tibia	Tarsus
**I**	*v*’	*d*, *(l)*, *v*’, *bv*”	*(l)*, *v*’, σ	*(l)*, *(v)*, φ_1_, φ_2_	*(ft)*, *pl*’, *(tc)*, *(it)*, *(p)*, *(u)*, *(a)*, *s*, *(pv)*, *(v)*, ε, ω_1_, ω_2_
**II**	*v*’	*d*, *(l)*, *v*”, *bv*”	*(l)*, σ	*(l)*, *(v)*, φ	*(ft)*, *(tc)*, *(it)*, *(p)*, *(u)*, *(a)*, *s*, *(pv)*, ω_1_, ω_2_
**III**	*d*, *l*’	*d*, *l*’, *ev*’	*l*’, σ	*l*’, *(v)*, φ	*(ft)*, *(tc)*, *(it)*, *(p)*, *(u)*, *(a)*, *s*, *(pv)*
**IV**	*v*’	*d*, *ev*’	*d*, *l*’	*l*’, *(v)*, φ	*ft*”, *(tc)*, *(p)*, *(u)*, *(a)*, *s*, *(pv)*

***Gender differences.*** No sexual dimorphism exists in external morphology, except for males being slightly smaller than females, their genital plates being slightly smaller proportionally than in females, and in the typical genitalic differences.

***Genetics*.** There are no other sequences of *Paraleius* or *Metaleius* on GenBank; however, GenBank blast searches of the COI sequence (KY402259) of *P. leahae* sp. n. generally matches that of other poronotic brachypyline oribatid mites. Further analysis was not performed.

##### Etymology.

This species is named after my wife and tireless supporter Leah Harper.

##### Remarks.

*Paraleius leahae* sp. n. is most similar to *P. leontonycha* (Travé 1960, Wunderle et al. 1990), which has been collected from under tree bark, in the galleries of bark beetles, and is phoretic on numerous species of bark beetles (Vitzthum 1926, Wunderle et al. 1990, Knee et al. 2013). *Paraleius leahae* sp. n. differs from *P. leontonycha* by having a long fusiform bothridium; monodactylous tarsi; presence of carina *kf*; solenidia of tibiae III and IV not microcephalic.

*Paraleius leahae* sp. n. differs from *P.* (=*Metaleius*) *strenzkei* in having a long fusiform bothridial seta; monodactylous tarsi, medial claw large and strongly hooked; carina *kf* present; total length (432–464) of *P. leahae* females greater than *P. strenzkei* (310–360) (Travé 1960).

According to Grandjean (1959) microcephalic solenidia are found only in arboricolous or saxicolous species. *Paraleius leontonycha*, *P. leahae* sp. n. and *P. strenzkei* are arboricolous species, the former has microcephalic solenidia and the latter two species lack this feature. The tips of solenidia on tibiae III and IV are delicate and prone to breakage, so it is possible that they are microcephalic in *P. leahae*; however, I examined more than 100 specimens without finding microcephalic tips.

### Distribution and biology

*Paraleius leontonycha* and *P. leahae* are quite similar morphologically, and it is possible that the latter has been misidentified as the former in the past. These two species are also ecologically similar in being corticolous and phoretic on bark beetles. The feeding biology of *P. leahae* and *P. leontonycha* is poorly understood, but fungal hyphae have been observed in the gut of slide mounted specimens of both species.

*Paraleius leontonycha* is the most commonly collected and widely distributed oribatid phoretic on bark beetles, however this species occurs infrequently and in low abundance (Norton 1980, Knee et al. 2013). *Paraleius leontonycha* has a Holarctic distribution; whereas, *P. leahae* has only been collected in Eastern Canada (Ontario, New Brunswick and Nova Scotia). *Paraleius* sp. and *P. leontonycha* collections at the CNC, SUNY-ESF, and the USDA were examined for *P. leahae* specimens. These collections contained material from across Canada (AB, BC, NB, NFLD, ON, QC), parts of the United States of America (AK, AZ, CA, LA, TX, UT, WI), parts of Europe (Croatia, Germany, Spain, Sweden, Switzerland), Mexico, Honduras, and Japan. All of the material examined from these collections represented *P. leontonycha*; no misidentified *P. leahae* were uncovered.

Typically the association between oribatid mites and their scolytine hosts is considered to be passive and with low host specificity (Norton 1980). *Paraleius leontonycha* is a host generalist, collected from 17 species of bark beetles (Knee et al. 2013, Ahadiyat and Akrami 2015). In contrast, *P. leahae* is a host specialist, collected from only two bark beetle species, *Hylastes porculus* and *Dendroctonus valens*. These two host species are not closely related species, but they are ecologically similar, as both species live in the stumps and roots of dead or dying conifers (Wood 1982). Multiple bark beetle species often occupy the same tree concurrently and occasionally their galleries cross, thus providing mites with an opportunity to transfer host species (Moser et al. 1971). *Paraleius leahae* shows a marked preference for only these two bark beetle species despite opportunities to switch host species. *Hylastes porculus* and *D. valens* are hosts to many species of mites; 16 other species of mites were collected from each of these host species in eastern Ontario including *P. leontonycha* (Knee et al. 2013). *Paraleius leahae* was the most abundant species collected out of the 33 species of mites collected from bark beetles in eastern Ontario using general lures (α-pinene and 95% ethanol) and Lindgren funnel traps (Knee et al. 2013). *Paraleius leahae* challenges the assumptions that bark beetle associated oribatid mites are uncommon and are not host specific.

### Key to known *Paraleius* species

**Table:** 

1	Tarsi monodactylous, central claw large sickle shaped and strongly hooked, hair-like lateral claws absent. Carina *kf* present. Long fusiform bothridial seta	***Paraleius leahae* sp. n.**
–	Tarsi hetero-tridactylous, large curved central claw, lateral claws hair-like. Carina *kf* absent. Capitate bothridial seta	**2**
2	Central claw sickle shaped and strongly hooked. Solenidia of tibiae III and IV microcephalic. Total length approximately 435–480 µm	***Paraleius leontonycha* (Berlese, 1910)**
–	Central claw evenly curved, c-shaped. Solenidia of tibiae III and IV not microcephalic. Total length approximately 310–360 µm	***Paraleius strenzkei* (Travé, 1960)**

## Supplementary Material

XML Treatment for Paraleius

XML Treatment for Paraleius leahae
